# In Vitro Ability of the Group B Streptococci to Inhibit Gram-Positive and Gram-Variable Constituents of the Bacterial Flora of the Female Genital Tract

**DOI:** 10.1155/S1064744995000391

**Published:** 1995

**Authors:** Pongsakdi Chaisilwattana, Gilles R. G. Monif

**Affiliations:** 1 Department of Obstetrics and Gynecology Faculty of Medicine Siriraj Hospital Mahidol University Bangkok Thailand; 2 Division of Infectious Diseases Department of Obstetrics and Gynecology Creighton University School of Medicine 601 North 30th Street, Ste 4700 Omaha NE 68131 USA

## Abstract

*Objective:* The purpose of this study was to analyze the ability of septicemic and nonsepticemic isolates of group B streptococci (GBS) to inhibit in vitro the principal bacterial groups found in the normal bacterial flora of the female genital tract.

*Methods:* The target groups were composed of 1) 10 strains each of the following: viridans streptococci, nonhemolytic streptococci (not group B or D), group A streptococci, GBS, peptostreptococci, coagulase-negative staphylococci, * Staphylococcus aureus*, and *Gardnerella vaginalis*; 2) 9 strains of enterococci; 3) 9 strains of group C or G streptococci; 4) 7 strains of lactobacilli; and 5) 7 strains of diphtheroids. All target groups were tested for inhibition by a test panel of either a group of 10 or 41 GBS isolates. If the GBS isolates failed to inhibit a target group, that group was tested for its ability to inhibit the GBS test panel.

*Results:* The GBS test panel did not inhibit the growth of coagulase-negative staphylococci or *S. aureus* but uniformly inhibited groups A, B, C, and G streptococci, lactobacilli, and *G. vaginalis*. One of the 7 strains of diphtheroids was inhibited by 37 of the 41 GBS isolates; the other 6 strains of diphtheroids were uniformly inhibited. Variable inhibition by GBS was observed with viridans streptococci, nonhemolytic (not group B or D) streptococci, peptostreptococci, and enterococci; however, inhibition or noninhibition was uniform for a given target strain against the entire GBS test panel. The 23 GBS isolates obtained from septicemic neonates or adults did not differ from the 18 nonsepticemic isolates in their ability to inhibit other species of streptococci or other gram-positive or gram-variable constituents of the bacterial flora of the female genital tract. When converse testing was done, all 10 GBS isolates were uniformly inhibited by coagulase-negative staphylococci and by the majority of enterococci, but were not inhibited by *S. aureus*.

*Conclusions:* These studies suggest that GBS may be significant regulators of other β-hemolytic streptococci, diphtheroids, lactobacilli, and *G. vaginalis* within the bacterial flora of the female genital tract. Moreover, the absence of GBS in the vaginal flora may be the result of mediation by coagulase-negative staphylococci and selected strains of enterococci.


Infectious Diseases in Obstetrics and Gynecology 3:91–97 (1995)
© 1995 Wiley-Liss, Inc.

### In Vitro Ability of the Group B Streptococci to Inhibit Gram-Positive and Gram-Variable Constituents of the Bacterial Flora of the Female Genital Tract

**Pongsakdi Chaisilwattana and Gilles R.G. Monif**

*Department of Obstetrics and Gynecology, Faculty of Medicine, Siriraj Hospital, Mahidol University, Bangkok, Thailand (P.C.); and Division of Infectious Diseases, Department of Obstetrics and Gynecology, Creighton University School of Medicine, Omaha, NE (G.R.G.M.)*

ABSTRACT

***Objective:*** The purpose of this study was to analyze the ability of septicemic and nonsepticemic isolates of group B streptococci (GBS) to inhibit in vitro the principal bacterial groups found in the normal bacterial flora of the female genital tract.

***Methods:*** The target groups were composed of 1) 10 strains each of the following: viridans streptococci, nonhemolytic streptococci (not group B or D), group A streptococci, GBS, peptostreptococci, coagulase-negative staphylococci, ***Staphylococcus aureus***, and ***Gardnerella vaginalis;*** 2) 9 strains of enterococci; 3) 9 strains of group C or G streptococci; 4) 7 strains of lactobacilli; and 5) 7 strains of diphtheroids. All target groups were tested for inhibition by a test panel of either a group of 10 or 41 GBS isolates. If the GBS isolates failed to inhibit a target group, that group was tested for its ability to inhibit the GBS test panel.

***Results:*** The GBS test panel did not inhibit the growth of coagulase-negative staphylococci or ***S. aureus*** but uniformly inhibited groups A, B, C, and G streptococci, lactobacilli, and ***G. vaginalis***. One of the 7 strains of diphtheroids was inhibited by 37 of the 41 GBS isolates; the other 6 strains of diphtheroids were uniformly inhibited. Variable inhibition by GBS was observed with viridans streptococci, nonhemolytic (not group B or D) streptococci, peptostreptococci, and enterococci; however, inhibition or noninhibition was uniform for a given target strain against the entire GBS test panel. The 23 GBS isolates obtained from septicemic neonates or adults did not differ from the 18 nonsepticemic isolates in their ability to inhibit other species of streptococci or other gram-positive or gram-variable constituents of the bacterial flora of the female genital tract. When converse testing was done, all 10 GBS isolates were uniformly inhibited by coagulase-negative staphylococci and by the majority of enterococci, but were not inhibited by ***S. aureus***.

***Conclusions:*** These studies suggest that GBS may be significant regulators of other β-hemolytic streptococci, diphtheroids, lactobacilli, and ***G. vaginalis*** within the bacterial flora of the female genital tract. Moreover, the absence of GBS in the vaginal flora may be the result of mediation by coagulase-negative staphylococci and selected strains of enterococci. © 1995 Wiley-Liss, Inc.

KEY WORDS

Bacterial interference, vaginal flora, streptococci strains

When quantitative and qualitative bacteriological studies are performed on the normal bacterial flora of the cervical and vaginal vault, the dominant aerobic groups of bacteria are lactobacilli, diphtheroids, staphylococci, streptococci, and occasionally members of Enterobacteriaceae.[1–6] The dominant anaerobic groups are composed of gram-positive bacilli which include lactobacilli, pep-

Address correspondence/reprint requests to Dr. Gilles R.G. Monif, Department of Obstetrics and Gynecology, Creighton University School of Medicine, 601 North 30th Street, Ste 4700, Omaha, NE 68131.

Received December 28, 1994

Accepted July 5, 1995

tostreptococci (which now incorporate peptococci), and Bacteroidaceae. Group B streptococci (GBS) constitute a potentially important subgroup within the streptococci. Not only are they frequent inhabitants of the bacterial flora of the female genital tract, but they are also the most common endogenous cause of monomicrobial disease for both parturitional gravidas and neonates.[7–10] The mechanisms that determine their presence, dominance, or exclusion are poorly delineated.

This study was carried out to analyze the ability of 23 isolates of GBS derived from septicemia patients and 18 isolates of GBS derived from vaginal specimens to inhibit other gram-positive or gram-variable bacteria that may be constituents of the bacterial flora of the female genital tract.

### MATERIALS AND METHODS

### Strains

The isolates of streptococci were provided by Microbiology Laboratory at St. Joseph Hospital, Omaha, NE; Gail Hill, Ph.D., Duke University School of Medicine, Durham, NC; Jon Rosenblatt, M.D., Mayo Clinic, Rochester, MN; Christine C. Sanders, Ph.D., Creighton University School of Medicine, Omaha, NE; and David F. Welch, M.D., University of Oklahoma College of Medicine, Oklahoma City, OK.

Initially, an inhibitor test panel of 41 isolates of GBS was examined. These inhibitor isolates were tested against target cultures of other streptococci and aerobic bacteria common in the vaginal flora. Twenty-three of the 41 isolates of GBS were obtained from blood cultures of septicemic patients. Of these 23 isolates, 15 were obtained from infants with early-onset or late-onset GBS disease, and the remaining were obtained randomly from other sources. Because of the uniformity of inhibition observed with the entire 41 isolates of GBS in the early experiments, the inhibitor test panel was subsequently reduced to 10 isolates of GBS. Of these 10 GBS isolates, 5 were derived from cases of early-onset neonatal septicemia and 5 were derived from incidental female-genital-tract cultures.

The target cultures included 10 viridans streptococci, 10 GBS, 10 nonhemolytic streptococci (not group B or D), 10 group A streptococci, 9 group C or G streptococci, 10 peptostreptococci, 9 enterococci, 10 coagulase-negative staphylococci, 10 *Staphylococcus aureus,* 7 lactobacilli, 7 diphtheroids, and 10 *Gardnerella vaginalis*. As an internal control, 10 target cultures of *Escherichia coli* were tested in 255 individual challenges to confirm the inability of GBS to inhibit gram-negative rods. The target sources of these cultures were vaginal isolates obtained in previous studies by one of the authors (G.R.G.M.).

### Media

Trypticase soy agar (TSA) (Baltimore Biological Laboratories, Baltimore, MD) was used for both layers in the overlay procedure. The organisms were maintained on TSA supplemented with 5% sheep blood (BAP, Scott Laboratories, Fiskeville, RI).

### Maintenance

All aerobic bacteria were subcultured to fresh BAP every 2 weeks, incubated for 24 h at 35°C in 10% $CO_2$ in air, and then held at 4°C. The anaerobic streptococci were grown under anaerobic conditions.

### Overlay Assay

A modification of the technique described by Fredericq[11] and further developed by Crow et al.[12] and Murray and Rosenblatt[13] was used for the overlay assays. Each strain of GBS was inoculated onto a 1-$cm^2$ area of a 15-ml TSA plate. Four strains per plate were tested. The organisms were incubated for 18–24 h in 10% $CO_2$ at 35°C. They were overlaid with 7.5 ml of molten TSA which was allowed to solidify. The target strain was then inoculated onto the top of the fresh TSA in the following manner. A 0.4 OD at 450 nm of the target strain was prepared in physiological saline. A 1:10 dilution was prepared in saline and a 2-ml quantity was inoculated onto the freshly overlaid plate. The excess was siphoned off, and the plates were incubated for 24 h at 35°C in 10% $CO_2$. The assays were performed in duplicate. After incubation, the assays were examined for inhibition of growth of the target strain (Fig. 1). The stab/chloroform technique was used for confirmation of inhibition.[12]

### RESULTS

### Viridans Streptococci

Seven strains of viridans streptococci were inhibited by all GBS strains examined in 101 tests (Table 1). Three viridans streptococci isolates were not

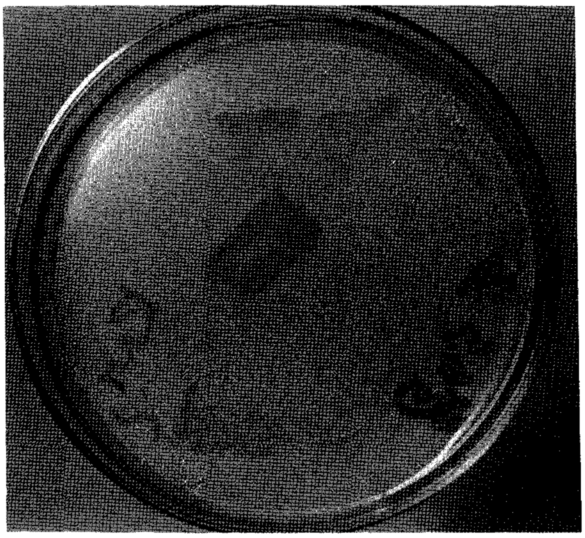

**Fig. 1. Demonstration of bacterial interference by GBS. The lawn of the target isolate shows inhibition of growth in the central area of streaking of the inhibitor strain underneath.**

inhibited by the GBS test panel. When inhibition was observed, the phenomenon was produced by the entire panel of 10 or 41 GBS.

### Nonhemolytic Streptococci (Not Group B or D)

Of the 10 target strains of the nonhemolytic streptococci (not group B or D), 9 isolates were inhibited by GBS. Comparable inhibition was produced by all of the GBS tested (Table 1).

### Enterococci

Of the 9 strains of enterococci, only 1 isolate was inhibited by GBS (Table 1). Although the results were uniform for both inhibition and noninhibition for the entire GBS panel, the degree of inhibition varied from isolate to isolate. When 5 strains of the enterococci were used as the inhibitor strain, all 10 isolates of the GBS tested in 50 challenge experiments were inhibited.

### Group A Streptococci

All 10 target strains of group A streptococci were inhibited by GBS in 193 challenge experiments (Table 1).

### GBS

For the 10 target strains of GBS, inhibition was complete in 193 challenge experiments (Table 1).

### Group C or G Streptococci

For the 9 challenge strains of group C (7) and group G (2) streptococci, inhibition was complete in all 183 challenge experiments (Table 1).

### Peptostreptococci

Of the 10 peptostreptococci, 7 challenge isolates were inhibited completely. Three of the 10 were not inhibited. The target isolates exhibited a uniform pattern of inhibition or noninhibition by GBS. The presence or absence of inhibition for the individual species of peptostreptococci is listed in Table 2.

### Coagulase-Negative Staphylococci

None of the 10 target isolates of coagulase-negative staphylococci tested in 193 individual challenge experiments was inhibited by GBS (Table 1). When 5 strains of coagulase-negative staphylococci were used as inhibitors, all 10 of the group of GBS isolates in 50 challenge experiments were inhibited.

### *S. aureus*

None of the 10 target isolates of *S. aureus* in 193 individual challenge experiments was inhibited by GBS (Table 1). When 5 strains of *S. aureus* were used as inhibitor cultures, none of the 10 GBS was inhibited in 50 challenge experiments.

### Lactobacilli

All 7 target isolates of lactobacilli were inhibited in 163 individual challenge experiments (Table 1).

### Diphtheroids

All 7 target isolates of diphtheroids tested individually were inhibited by GBS. One isolate had a variable pattern of inhibition so that, of the 194 individual experiments, 190 showed inhibition (Table 1).

### *G. vaginalis*

All 10 target isolates of *G. vaginalis* were inhibited by GBS in 193 individual challenge experiments (Table 1).

### DISCUSSION

The initial concept of bacterial interference emanated from the observations of Pasteur and Joubert.[14] They noted that *Bacillus anthracis* in urine cultures would die if contaminated by other

TABLE 1. Inhibition of target bacteria by GBS isolates

| Target bacteria | No. of strains tested | No. of observations | No. of strains/ (No. of observations) | |
|---|---|---|---|---|
| | | | Inhibited | Noninhibited |
| Viridans streptococci | 10 | 193 | 7/(101) | 3/(92) |
| Nonhemolytic streptococci (not group B or D) | 10 | 193 | 9/(183) | 1/(10) |
| Enterococci | 9 | 276 | 1/(41) | 8/(235) |
| Group A streptococci | 10 | 193 | 10/(193) | 0/(193) |
| GBS | 10 | 193 | 10/(193) | 0/(193) |
| Group C (7) or G (2) streptococci | 9 | 183 | 9/(183) | 0/(183) |
| Peptostreptococci | 10 | 193 | 7/(132) | 3/(61) |
| Coagulase-negative staphylococci | 10 | 193 | 0/(193) | 10/(193) |
| *S. aureus* | 10 | 193 | 0/(193) | 10/(193) |
| Lactobacilli | 7 | 163 | 7/(163) | 0/(163) |
| Diphtheroids | 7 | 193 | 7/(186)[a] | 0/(4) |
| *G. vaginalis* | 10 | 193 | 10/(193) | 0/(193) |

[a]Four of GBS in the panel of 1 isolate were inhibitory.

TABLE 2. In vitro bacterial interference by GBS on 10 strains of peptostreptococci

| Individual peptostreptococcal isolates | No. of test strains of GBS | % Inhibition |
|---|---|---|
| *P. tetradius* | 10 | 100 |
| | 10 | 0 |
| *P. anaerobius* | 41 | 100 |
| | 10 | 100 |
| | 10 | 100 |
| *P. micros* | 41 | 100 |
| | 10 | 0 |
| *P. asaccharolyticus* | 41 | 0 |
| | 10 | 100 |
| | 10 | 100 |

bacteria. The mechanisms by which a bacterial species maintains its ecological niche are varied. Inhibitor bacterial products include a wide range of substances: low-molecular-weight antibiotics, metabolic products, hydrogen peroxide, lytic agents, enzymes, bacteriocins, and bacteriophages.[15,16] The ultimate question for GBS is how this normal constituent of the bacterial flora of the female genital tract survives or governs. These studies[15,16] demonstrate that GBS have the ability, through bacterial interference, to defend their ecological niches in vitro, not only against other GBS but also against β-hemolytic strains of group A, C, and G streptococci. This ability appears to be uniform, which may be the result of a genetic interrelationship between hemolytic activity and bacterial interference. Brock et al. found that, by categorizing strains of *S. zymogens* in terms of their hemolytic character, they could demonstrate uniform bacterial interference mediated by bacteriocins.[17] In their study, they found no variation in the ability of GBS to inhibit bacterial replication between septicemic and nonsepticemic GBS isolates. In our study, no differences in inhibition or noninhibition were identified between septicemic isolates from incidental vaginal cultures. The primary risk factors that account for a statistically significant increase in the anticipated incidence of GBS diseases in neonates are related to their ability to colonize the urinary tract (bacteriuria) and to achieve high-density replication within the vaginal and rectal bacterial flora.[18]

Based on preliminary observations, other investigators have reported that heavy-density colonization is due primarily to the avid ability of selected strains of beta hemolytic streptococci to adhere to genitourinary epithelial cells rather than to a unique ability to regulate the associated vaginal flora[19], Reed et al. looked at group A streptococcal adherence to pharyngeal cells in isolates from cases of acute rheumatic fever isolates.[20] They found that streptococci strains associated with acute rheumatic fever appeared to adhere more avidly to pharyngeal cells than strains not associated with rheumatic fever.

The potential of GBS to govern the enterococci is significant. The majority of isolates (95%) exhibited complete inhibition. The impact of GBS on

NO INHIBITION

Enterobacteriaceae
Coagulase-negative staphylococci
*Staphylococcus aureus*

COMPLETE INHIBITION

Other group B streptococci

GROUP B
STREPTOCOCCI

Group A, C, G streptococci
*Gardnerella vaginalis*
lactobacilli
diphtheroids

MINORITY OF STRAINS
INHIBITED
Enterococci

MAJORITY OF
STRAINS INHIBITED
viridans streptococci (70%)

Non-B, non-D
streptococci exhibiting
no hemolysis (90%)

Peptostreptococci (63%)

Fig. 2. Schematic representation of the ability of GBS to inhibit replication of streptococci and nonstreptococcal aerobic bacteria endogenous to the bacterial flora of the female genital tract.

viridans streptococci, enterococci, and peptostreptococci was significantly less. GBS inhibited other common nonstreptococcal gram-positive aerobic bacteria and *G. vaginalis* but had no impact on staphylococci. These observations, along with those of other studies in the literature, may provide insight regarding the bacterial interrelationships within the bacterial flora of the female genital tract.

Traditionally, the dominance of lactobacilli has been thought to correlate with the normality of the vaginal bacterial flora. De Klerk and Coetzec studied bacterial inhibition by lactobacilli. Using supernatants concentrated by ammonium-sulfate precipitators, they were able to demonstrate an antibacterial spectrum that was primarily restricted to certain members of the family Lactobacteriaceae.[21] A significant number of enterococci were inhibited. The antibiotic-like supernatants had no impact upon the Enterobacteriaceae or staphylococci. Holmberg and Hallander documented the ability of *Streptococcus sanguis* to inhibit *Lactobacillus acidophilus, L. fermentum,* and *L. casei.*[22] Phonck, among others, also demonstrated the ability of streptococci to inhibit vaginal lactobacilli.[23] The importance of lactobacilli may be more their role as regulators of enterococci than as major regulators of GBS.

Statistically, coagulase-negative staphylococci are more frequently present in the bacterial flora than *Staphylococcus aureus*.[1–4] Both coagulase-negative and coagulase-positive staphylococci have significant ability to inhibit other bacteria. Possibly more important is their insensitivity to bacterial interference by other constituents of the bacterial flora. Dajani and Wannamaker[24] demonstrated the ability of *S. aureus* to produce a bactericidal substance that inhibits group A, D, and G streptococci. Observations in clinical disease in which both staphylococci and β-hemolytic streptococci can be concomitantly isolated from skin lesions have raised questions as to whether staphylococci invade sites previously infected with β-hemolytic organisms or a significant coupling occurs between the 2 groups of gram-positive bacteria.[24–27] Theoretically, *S. aureus* as the dominant staphylococcal species may occur either directly (by insensitivity to bacterial interference) or indirectly. Anaerobic bacteria (particularly *Bacteroides melaninogenicus* and *B. fragilis*)

can counter the inhibition of coagulase-negative staphylococci, thereby allowing *S. aureus* to occupy the void.

The ability of a given strain of Enterobacteriaceae to inhibit other members of the family has been well documented.[16] The predominance of a strain of *E. coli* as the principal Enterobacteriaceae in the bacterial flora may also be the result of Bacteroidaceae's inhibition of competing strains. Murray and Rosenblatt[13] demonstrated that *B. melaninogenicus*, *B. fragilis*, and *B. oralis*, while possessing significant ability to inhibit *Enterobacter cloacae*, *E. aerogenes*, *Klebsiella* species, and *Serratia marcescens*, were ineffective against *E. coli* and *Morganella morganii*. Bacteroidaceae had moderate inhibitor activity against coagulase-negative staphylococci but almost no activity against *S. aureus*. In their report, fusobacteria and *L. fermentum* had little inhibitory effect on either gram-negative or gram-positive bacteria. Interspecies governance among the Enterobacteriaceae is probably mediated by bacteriocins, but the predominance of *E. coli* and *Proteus mirabilis* may be a direct function of their resistance to bacterial inhibition by Bacteroidaceae.

Our demonstration of the in vitro ability of GBS to inhibit streptococci, lactobacilli, diphtheroids, *G. vaginalis*, and most hemolytic and nonhemolytic streptococci infers that GBS may be significant regulators of the bacterial flora of the female genital tract (Fig. 2). The presence of GBS in the vaginal flora may be determined by the absence of coagulase-negative staphylococci or selected strains of enterococci. The studies of bacterial inhibition in the literature, coupled with the present observations, infer that the ability of GBS to participate in progressive polymicrobial anaerobic infection may result as a consequence of the inhibition of coagulase-negative staphylococci by Bacteroidaceae.[28]

### REFERENCES

1. Bartlett JG, Moon NE, Goldstein PR, et al.: Cervical and vaginal bacterial flora: Ecologic niches in the female lower genital tract. Am J Obstet Gynecol 130:658–661, 1978.
2. Gorbach SL, Mend KB, Thadepalli H, et al.: Anaerobic microflora of the cervix in healthy women. Am J Obstet Gynecol 117:1053–1055, 1973.
3. Ohm MJ, Galask RP: Bacterial flora of the cervix from 100 prehysterectomy patients. Am J Obstet Gynecol 122: 683–687, 1975.
4. Tashjiar JH, Coulam CB, Washington JA II: Vaginal flora in asymptomatic women. Mayo Clin Proc 51:557–561, 1976.
5. Levison ME, Corman LC, Carrington ER, et al.: Quantitative microflora of the vagina. Am J Obstet Gynecol 127:80–85, 1987.
6. Monif GRG, Thomson JL, Stephens HG, et al.: Quantitative and qualitative effects of providone-iodine liquid and gel on the aerobic and anaerobic flora of the female genital tract. Am J Obstet Gynecol 137:432–438, 1980.
7. Baker CJ, Barrett FR: Transmission of group B streptococci among parturient women and their neonates. J Pediatr 83:919–925, 1973.
8. Berquist G, Hurvell B, Malmborg AS, et al.: Neonatal infections caused by group B streptococcus. Scand J Infect Dis 3:209–212, 1971.
9. Ledger WJ, Norman M, Gee C, et al.: Bacteremia on an obstetric-gynecology service. Am J Obstet Gynecol 121: 205–212, 1975.
10. White CA, Koontz FP: Group B hemolytic streptococcus infections in postpartum patients. Obstet Gynecol 41:27–32, 1973.
11. Fredericq R: Action antibiotiques reciproques chez les Enterobacteriaceae. Rev Belge Pathol Med Exp Suppl 4:1–40, 1948.
12. Crowe CC, Sanders WE, Longley S: Bacterial interference. II. Role of the normal throat flora in prevention of colonization by group A streptococcus. J Infect Dis 128: 527–532, 1973.
13. Murray PR, Rosenblatt JE: Bacterial interference by oropharyngeal and clinical isolates of anaerobic bacteria. J Infect Dis 134:281–285, 1976.
14. Pasteur L, Joubert JF: Charbon et septicemie. C R Soc Bio Paris 85:101–117, 1877.
15. Tagg JR, Dajani AS, Wannamaker LW: Bacteriocin of gram-positive bacteria. Bacteriol Rev 40:722–756, 1976.
16. Reeves R: The bacteriocins. Bacteriol Rev 29:25–45, 1965.
17. Brock TD, Peacher B, Pierson D: Survey of the bacteriocins of enterococci. J Bacteriol 86:702–707, 1963.
18. Monif GRG (ed): Group B Streptococci in Infectious Diseases in Obstetrics and Gynecology. 3rd ed. Omaha: Parthenon Publications, pp 260–270, 1994.
19. Reed WP, Williams RC Jr: Bacterial adherence: First step in pathogenesis of certain infections. J Chron Dis 31:67–72, 1978.
20. Reed WP, Selinger DS, Albright EL, et al.: Streptococcal adherence to pharyngeal cells of children with acute rheumatic fever. J Infect Dis 142:803–810, 1980.
21. De Klerk HC, Coetzec JM: Antibiosis among lactobacilli. Nature 192:340–341, 1961.
22. Holmberg K, Hallander HO: Interference between gram-positive microorganisms in dental plaque. J Dent Res 51:588–595, 1972.
23. Phonck M: Streptococci antagonizing the vaginal lactobacillus. J Hung Epidemiol Microbiol Immunol (Prague) 3:267–270, 1962.
24. Dajani A, Wannamaker LW: Demonstration of a bactericidal substance against beta-hemolytic streptococci in supernatant of fluids of staphylococcal cultures. J Bacteriol 97:985–991, 1969.
25. Anthony BF, Wannamaker LW: Bacterial interference in experimental burns. J Exp Med 125:319–336, 1967.
26. Ribble JC: A mechanism of bacterial interference in vitro. J Immunol 98:716–723, 1967.
27. Shinefield HR, Ribble JC, Eichenwald JF, et al.: Bacterial interference: Its effect on nursery-acquired infections with *S. aureus*. Am J Dis Child 105:683–688, 1963.
28. Monif GRG (ed): Infectious Diseases in Obstetrics and Gynecology. 3rd ed. Omaha: IDI Publications, 1993.
